# The sodium-potassium pump is an information processing element in brain computation

**DOI:** 10.3389/fphys.2014.00472

**Published:** 2014-12-23

**Authors:** Michael D. Forrest

**Affiliations:** Department of Computer Science, University of WarwickCoventry, UK

**Keywords:** sodium-potassium pump, Purkinje cell, neuron model, cerebellum, neural code, Na^+^/K^+^ pump, Na^+^/K^+^-ATPase

Brain neurons can transmit signals using a flow of Na^+^ and K^+^ ions, which produce an electrical spike called an action potential (AP) (Hodgkin and Huxley, 1952). After an AP, the Na^+^/K^+^ pump resets the arrangement of Na^+^ and K^+^ ions back to their original positions so that the neuron is then ready to relay another AP when it is called upon to do so (Glitsch, 2001). So, the Na^+^/K^+^ pump has a “housekeeping” role rather than a direct role in brain signaling. This is the long-held, entrenched viewpoint. However, novel research upon cerebellar Purkinje neurons suggests that the Na^+^/K^+^ pump may have a direct role in brain coding and computation (Forrest, 2008, 2014a,b; Forrest et al., 2009, 2012). This research was conducted in 2006–2007, and presented in a 2008 Ph.D. thesis (Forrest, 2008), but has only been published relatively recently. In the intervening period it was serially rejected by reviewers and journals that were uncomfortable with this re-appraisal of Na^+^/K^+^ pump function. Purkinje neurons are found in the cerebellum, responsible for motor control (Ito, 1984).

The Na^+^/K^+^ pump uses the energy of one ATP molecule to exchange three intracellular Na^+^ ions for two extracellular K^+^ ions (Glitsch, 2001). Thus, the pump is electrogenic, extruding one net charge per cycle to hyperpolarize the membrane potential. *In vitro*, the Na^+^/K^+^ pump has been shown to control and set the intrinsic activity mode of Purkinje neurons (Forrest, 2008, 2014a; Forrest et al., 2009, 2012). It dictates whether the Purkinje neuron is quiescent or spontaneously firing in a continuous tonic, continuous burst, bimodal (tonic and quiescent), trimodal (tonic, burst, quiescent), or bimodal (burst and quiescent) repeat pattern. In the bimodal and trimodal repeat patterns, the Na^+^/K^+^ pump sets the length of each constituent mode. So, at the foundation of the Purkinje cell's intrinsic multimodality, there is the working of just a single molecular species: the Na^+^/K^+^ pump.

Numerical modeling of experimental data suggests that, *in vivo*, the Na^+^/K^+^ pump produces long quiescent punctuations (>>1 s) to Purkinje neuron firing (Forrest, 2014a). The Na^+^/K^+^ pump is an enzyme and its activity is dependent on the concentration of its substrates: intracellular Na^+^ and extracellular K^+^ (Glitsch, 2001). Na^+^ flows into and accumulates in the Purkinje cell during firing; Forrest's numerical model proposes that intracellular Na^+^ concentration is a memory element, which records firing history (Forrest, 2014a). Furthermore, that the Na^+^/K^+^ pump “reads” this memory setting to dictate the timing and duration of long quiescent periods. To speculate, these long quiescent periods, on the scale of seconds and minutes, may be computationally advantageous. By conferring an access to longer time scales, they may permit storage and short-term processing of sensory information in the cerebellar cortex. To elaborate, they may permit different dynamical states to be sustained in the cerebellar cortex for extended periods. Each of these states is associated with a specific configuration of firing and quiescent states in different Purkinje cells. These network states could store information and perform computations. So, these network computations sit upon the proposed intracellular Na^+^ ion computation, mediated by the Na^+^/K^+^ pump, which dictates the activity state of individual Purkinje neurons (firing or quiescent). Forrest terms this hypothesis: “ion to network” computation (Forrest, 2014a). There could be further layers of control and regulation: the Na^+^/K^+^ pump is a receptor for the endo-ouabain signaling molecule (Xie and Cai, 2003) and Na^+^/K^+^ pump activity might be modulated by intracellular signaling cascades (Therien and Blostein, 2000; Bagrov and Shapiro, 2008). Relevantly, a mutation in the Na^+^/K^+^ pump causes rapid onset dystonia parkinsonism, which has symptoms to indicate that it is a pathology of cerebellar computation (Cannon, 2004; de Carvalho et al., 2004). Furthermore, an ouabain block of Na^+^/K^+^ pumps in the cerebellum of a live mouse results in it displaying movement disorders: ataxia and dystonia (Calderon et al., 2011).

In recent times, other groups have shown the Na^+^/K^+^ pump to be a computational element in other neuron types, in other systems. For example, the Na^+^/K^+^ pump produces an afterhyperpolarization (AHP) to each burst in the motor neurons of Drosophilia larvae; AHP amplitude is dependent on the number of spikes in the burst (Glanzman, 2010; Pulver and Griffith, 2010). So the Na^+^/K^+^ pump generated AHP acts as a spike counter and is a form of short-term memory.

Locomotion in Xenopus tadpoles, as in vertebrates generally, is produced by a central pattern generator (CPG) network within the animal's spinal cord and hindbrain. In these CPG neurons the Na^+^/K^+^ pump produces an AHP; the amplitude/hyperpolarization (and therefore duration) of which is proportional to the intensity and duration of previous spiking activity (Simmers, 2012; Zhang and Sillar, 2012). So, again, a Na^+^/K^+^ pump generated AHP acts as a spike counter and a form of short-term memory. This system ensures that if the tadpole has recently swum intensively (many spikes per unit time) the next swimming bout is shorter; whereas if prior swimming was weaker (less spikes) then the next swimming bout can be longer. This presumably ensures that the animal is not overexerted. So, the Na^+^/K^+^ pump generated AHP system remembers prior spiking/motor activity to dictate the nature of future spiking/motor activity.

Aplysia interneurons can exhibit a prolonged inhibitory synaptic potential, produced by synaptic activation of an electrogenic Na^+^ pump (Pinsker and Kandel, 1969). In sensory neurons of the lamprey, and tactile (T) sensory neurons of the leech, the Na^+^/K^+^ pump produces an AHP (Baylor and Nicholls, 1969; Jansen and Nicholls, 1973; Van Essen, 1973; Catarsi and Brunelli, 1991; Catarsi et al., 1993; Parker et al., 1996; Scuri et al., 2007). Activation of a leech's T neurons produces swimming behavior; serotonin quickens the swimming response to touch (reaction time) (Scuri et al., 2007). It negatively modulates Na^+^/K^+^ pumping, leading to less AHP, and more spikes being transmitted across a synaptic connection between two T neurons i.e., there results a functional, plastic change at a synapse (learning).

The leech T neuron responds to step-stimuli on the skin with a burst code (Arganda et al., 2007). The code doesn't seem to contain any amplitude information, only the velocity of the skin displacement. With Gaussian white-noise stimuli, stimulus velocity is encoded in burst duration. The greater the velocity, the longer the burst (and the greater the spike rate within the burst). However, a given velocity does not always produce the same length of burst; the length depends on the nature of other velocities coded for in the same period. The burst length codes the ratio of that velocity to the standard deviation of the velocity distribution. This means that a burst of given size is first produced in response to low velocities, but after 1 min of stimulation it is only produced in response to higher velocities i.e., there is adaptation to the stimulus. Numerical simulations and experiments indicate that the Na^+^/K^+^ pump is responsible for this adaptive scaling. When the pump is blocked by strophantidin it cannot occur. Under normal conditions: the larger the stimulus velocity variance, the greater the AHP amplitude and, with this decreased excitability, bursts are smaller.

Forrest and co-workers have shown, by experiment and modeling, that the Na^+^/K^+^ pump can produce AHPs in cerebellar Purkinje neurons (Forrest, 2008, 2014a,b; Forrest et al., 2009, 2012). Other groups have shown that the Na^+^/K^+^ pump can produce AHPs in other mammalian neurons: in presynaptic and postsynaptic neurons at the calyx of Held in the medial nucleus of the trapezoid body (Kim et al., 2007; Kim and von Gersdorff, 2012), in spinal cord neurons (Ballerini et al., 1997), in cerebellar Golgi cells (Botta et al., 2010), in hippocampal pyramidal (Gustafsson and Wigström, 1981, 1983; Thompson and Prince, 1986; Gulledge et al., 2013), and in neocortical layer 5 (Koike et al., 1972; Gulledge et al., 2013) neurons.

In rats, small-diameter neurons of the dorsal root ganglion (DRG) secrete follistatin-like 1 (FSTL1) at their axon synapse, in a spontaneous and depolarization dependent manner. This FSTL1 feeds back and activates their hyperpolarizing Na^+^/K^+^ pumps (Li et al., 2011). This reduces their excitability and their excitatory transmission to downstream neurons. This appears to be a feedback loop, which keeps the transmission at this synapse in the correct range.

Interestingly, Na^+^/K^+^ pump generated AHPs can be observed in canine coronary sinus fibers (Wit et al., 1981). What is their role in these cardiac cells? Are they are a mechanism to prevent over-excitation (and perhaps arrhythmias)? Or are they without function here; are Na^+^/K^+^ pump generated AHPs a nuanced by-product of how some excitable cells work, which certain neural cells have seized to their advantage for coding strategies?

There is not likely a single, definitive neural code; different neuron types probably employ different strategies. We have discussed how some neurons, in some systems, utilize the Na^+^/K^+^ pump. However, for some other neurons, Na^+^/K^+^ pump activity doesn't seem to be a major determinant of their firing pattern. For example, with a substantial proportion of Na^+^/K^+^ pumps blocked by ouabain, the rat motor neuron shows no appreciable change in firing pattern or rate (Sawczuk et al., 1997). By contrast, a much smaller ouabain concentration can produce a dramatic sequence of changes in the firing pattern of cerebellar Purkinje neurons (Forrest et al., 2012). Impaired Na^+^/K^+^ pump function in Drosophila (Ashmore et al., 2009), through mutation or ouabain application, doesn't appear to universally corrupt all brain functions but a specific few: again, suggesting that only some—and not all—neuron types employ it in a coding role. A complicating factor in interpreting these experiments is that there can be different Na^+^/K^+^ pump isoforms in different cell types in different species and these can have different ouabain sensitivities (the Na^+^/K^+^ pump is made up of an α and a β subunit; four isoforms of the α and three of the β are known and these can admix in different combinations; Blanco and Mercer, 1998).

In this paper we have listed a number of systems in which the Na^+^/K^+^ pump may be directly involved in computation. To repeat the case for the cerebellar Purkinje neuron (Forrest, 2014a); electrogenic Na^+^/K^+^ pump activity hyperpolarises the membrane potential, acting to drive its value more negative, and thus it directly inputs and contributes to the computational variable of the brain cell: its membrane voltage. Pump activity is enzymatically dependent upon intracellular Na^+^ and extracellular K^+^ concentrations, which are a record of prior membrane voltage values as they are a function of voltage-dependent ion conductances. So, Na^+^/K^+^ pump “reads” membrane voltage history to shape future membrane voltages. It mediates a form of cellular memory. Its activity can dramatically change the membrane potential; for example, switching off spiking to render a long period of hyperpolarized quiescence (>> 1 s). Indeed, it can act as a spike integrator, memorizing past firing activity to dictate the timing, amplitude, and duration of quiescent periods (AHPs). So, information may be encoded in quiescent periods, in addition to spiking parameters. This may be especially salient for inhibitory neurons, where quiescence conveys disinhibition to downstream neurons. For example, Purkinje cells provide inhibitory input to the Deep Cerebellar Nuclei (DCN). Quiescent periods may be important in cerebellar functioning because numerous Purkinje neurons converge upon and inhibit a single DCN neuron (~40:1). If all these Purkinje neurons are continuously, simultaneously active then it might be that the DCN neuron is unable to fire in any meaningful way. With a proportion of the Purkinje cells quiescent, only a fraction of the population is active and relevant. The members making up this relevant subset can be switched and changed in a controlled fashion, which can be utilized as a computational feature.

In summary, there is a growing body of work suggesting that Na^+^/K^+^ pumps can sub-serve information processing roles in some neurons. This is perhaps not surprising given that time and again evolution has shown herself to be a master of turning cost to advantage. Na^+^/K^+^ pumping in the brain accounts for the overwhelming majority of an animal's energy (ATP) consumption (Laughlin et al., 1998; Attwell and Laughlin, 2001); it seems that the brain may have furthered what information it can process from this cost in some neurons.

## Conflict of interest statement

The author declares that the research was conducted in the absence of any commercial or financial relationships that could be construed as a potential conflict of interest.

## References

[B1] Arganda S. Guantes R. de Polavieja G. G. (2007). Sodium pumps adapt spike bursting to stimulus statistics. Nat. Neurosci. 11, 1467–1473. 10.1038/nn1982 17906619

[B2] Ashmore L. J. Hrizo S. L. Paul S. M. Van Voorhies W. A. Beitel G. J. Palladino M. J. (2009). Novel mutations affecting the Na, K ATPase alpha model complex neurological diseases and implicate the sodium pump in increased longevity. Hum. Genet. 126, 431–447. 10.1007/s00439-009-0673-2 19455355 PMC2791699

[B3] Attwell D. Laughlin S. B. (2001). An energy budget for signaling in the grey matter of the brain. J. Cereb. Blood Flow Metab. 21, 1133–1145. 10.1097/00004647-200110000-00001 11598490

[B4] Bagrov A. Y. Shapiro J. I. (2008). Endogenous digitalis: pathophysiologic roles and therapeutic applications. Nat. Clin. Pract. Nephrol. 4, 378–392. 10.1038/ncpneph0848 18542120 PMC2574729

[B5] Ballerini L. Bracci E. Nistri A. (1997). Pharmacological block of the electrogenic sodium pump disrupts rhythmic bursting induced by strychnine and bicuculline in the neonatal rat spinal cord. J. Neurophysiol. 77, 17–23. 9120558 10.1152/jn.1997.77.1.17

[B6] Baylor D. A. Nicholls J. G. (1969). After-effects of nerve impulses on signalling in the central nervous system of the leech. J. Physiol. (Lond.) 203, 571–589. 5387027 10.1113/jphysiol.1969.sp008880 PMC1351531

[B6a] Blanco G. Mercer R. W. (1998). Isozymes of the Na-K-ATPase: heterogeneity in structure, diversity in function. Am. J. Physiol. 275(5 Pt 2), F633–F650. 9815123 10.1152/ajprenal.1998.275.5.F633

[B7] Botta P. de Souza F. M. S. Sangrey T. De Schutter E. Valenzuela C. F. (2010). Alcohol excites cerebellar golgi cells by inhibiting the Na+;/K+ ATPase. Neuropsychopharmol 35, 1984–1996. 10.1038/npp.2010.76 20520600 PMC2904864

[B8] Calderon D. P. Fremont R. Kraenzlin F. Khodakhah K. (2011). The neural substrates of rapid-onset dystonia-parkinsonism. Nat. Neurosci. 14, 357–365 10.1038/nn.2753 21297628 PMC3430603

[B9] Cannon C. (2004). Paying the price at the pump: dystonia from mutations in a Na^+^/K^+^-ATPase. Neuron 43, 153–154. 10.1016/j.neuron.2004.07.002 15260948

[B10] Catarsi S. Brunelli M. (1991). Serotonin depresses the after-hyperpolarization through the inhibition of the Na+/K+ electrogenic pump in T sensory neurones of the leech. J. Exp. Biol. 155, 261–273. 1849955 10.1242/jeb.155.1.261

[B11] Catarsi S. Scuri R. Brunelli M. (1993). Cyclic AMP mediates inhibition of the Na (+)-K+ electrogenic pump by serotonin in tactile sensory neurones of the leech. J. Physiol. (Lond.) 462, 229–242. 7687293 10.1113/jphysiol.1993.sp019552 PMC1175298

[B12] de Carvalho A. P. Sweadner K. J. Penniston J. T. Zaremba J. Liu L. Caton M.. (2004). Mutations in the Na^+^/K^+^-ATPase [alpha]3 gene ATP1A3 are associated with rapid-onset dystonia parkinsonism. Neuron 43, 169–175. 10.1016/j.neuron.2004.06.028 15260953

[B13] Forrest M. D. (2008). The Biophysics of Purkinje Computation. Ph.D. thesis, University of Warwick.

[B14] Forrest M. D. (2014a). Intracellular calcium dynamics permit a Purkinje neuron model to perform toggle and gain computations upon its inputs. Front. Comput. Neurosci. 8:86. 10.3389/fncom.2014.00086 25191262 PMC4138505

[B16a] Forrest M. D. (2014b). Biophysics and Computations of the Cerebellar Purkinje Neuron. Seattle, WA: Createspace.

[B15] Forrest M. D. Wall M. J. Press D. A. (2009). The sodium-potassium pump controls the intrinsic firing of the cerebellar Purkinje neuron, in Poster Session Presented at: 16^th^ International Conference on Neural Information Processing (ICONIP 09), (Bangkok). 10.1371/journal.pone.0051169 PMC3527461 23284664

[B16] Forrest M. D. Wall M. J. Press D. A. Feng J. (2012). The sodium-potassium pump controls the intrinsic firing of the cerebellar Purkinje neuron. PLoS ONE 7:e51169. 10.1371/journal.pone.0051169 23284664 PMC3527461

[B17] Glanzman D. L. (2010). Ion pumps get more glamorous. Nat. Neurosci. 13, 4–5. 10.1038/nn0110-4 20033078

[B18] Glitsch H. G. (2001). Electrophysiology of the sodium-potassium-ATPase in cardiac cells. Physiol. Rev. 81, 1791–2826. 11581502 10.1152/physrev.2001.81.4.1791

[B19] Gulledge A. T. Dasari S. Onoue K. Stephens E. K. Hasse J. M. Avesar D. (2013). A sodium-pump-mediated afterhyperpolarization in pyramidal neurons. J. Neurosci. 33, 13025–13041. 10.1523/JNEUROSCI.0220-13.2013 23926257 PMC3735883

[B20] Gustafsson B. Wigström H. (1981). Evidence for two types of afterhyperpolarization in CA1 pyramidal cells in the hippocampus. Brain Res. 206, 462–468. 10.1016/0006-8993(81)90548-5 6260282

[B21] Gustafsson B. Wigström H. (1983). Hyperpolarization following long-lasting tetanic activation of hippocampal pyramidal cells. Brain Res. 275, 159–163. 10.1016/0006-8993(83)90429-8 6313125

[B22] Hodgkin A. L. Huxley A. F. (1952). A quantitative description of membrane current and its application to conduction and excitation in nerve. J. Physiol. (Lond.) 117, 500–544. 12991237 10.1113/jphysiol.1952.sp004764 PMC1392413

[B23] Ito M. (1984). The Cerebellum and Neural Control. New York, NY: Raven.

[B24] Jansen J. K. S. Nicholls J. G. (1973). Conductance changes, an electrogenic pump and the hyperpolarization of leech neurones following impulses. J. Physiol. (Lond.) 229, 635–655. 4693676 10.1113/jphysiol.1973.sp010158 PMC1350554

[B25] Kim J. H. Sizov I. Dobretsov M. von Gersdorff H. (2007). Presynaptic Ca2 buffers control the strength of a fast post-tetanic hyperpolarization mediated by the alpha3 Na^+^/ K^+^-ATPase. Nat. Neurosci. 10, 196–205. 10.1038/nn1839 17220883

[B26] Kim J. H. von Gersdorff H. (2012). Suppression of spikes during posttetanic hyperpolarization in auditory neurons: the role of temperature, Ih currents, and the Na+-K+-ATPase pump. J. Neurophysiol. 108, 1924–1932. 10.1152/jn.00103.2012 22786951 PMC3545002

[B27] Koike H. Mano N. Okada Y. Oshima T. (1972). Activities of the sodium pump in cat pyramidal tract cells investigated with intracellular injection of sodium ions. Exp. Brain Res. 14, 449–462. 10.1007/BF00236587 5047281

[B28] Laughlin S. B. de Ruyter van Steveninck R. R. Anderson J. C. (1998). The metabolic cost of neural information. Nat. Neurosci. 1, 36–41. 10.1038/236 10195106

[B29] Li K. C. Zhang F. X. Li C. L. Wang F. Yu M. Y. Zhong Y. Q.. (2011). Follistatin-like 1 suppresses sensory afferent transmission by activating Na^+^, K^+^-ATPase. Neuron 69, 974–987 10.1016/j.neuron.2011.01.022 21382556

[B30] Parker D. Hill R. Grillner S. (1996). Electrogenic pump and a Ca2+-dependent K+ conductance contribute to a posttetanic hyperpolarization in lamprey sensory neurons. J. Neurophysiol. 76, 540–553. 8836242 10.1152/jn.1996.76.1.540

[B31] Pinsker H. Kandel E. R. (1969). Synaptic activation of an electrogenic sodium pump. Science 163, 931–935. 10.1126/science.163.3870.931 5763875

[B32] Pulver S. R. Griffith L. C. (2010). Spike integration and cellular memory in a rhythmic network from Na^+^/K^+^ pump current dynamics. Nat. Neurosci. 13, 53–59. 10.1038/nn.2444 19966842 PMC2839136

[B33] Sawczuk A. Powers R. K. Binder M. D. (1997). Contribution of outward currents to spike-frequency adaptation in hypoglossal motoneurons of the rat. J. Neurophysiol. 78, 2246–2253. 9356378 10.1152/jn.1997.78.5.2246

[B34] Scuri R. Lombardo P. Cataldo E. Ristori C. Brunelli M. (2007). Inhibition of Na^+^/K^+^ ATPase potentiates synaptic transmission in tactile sensory neurons of the leech. Eur. J. Neurosci. 25, 159–167. 10.1111/j.1460-9568.2006.05257.x 17241277

[B35] Simmers J. (2012). Motor control: learning new moves with old pumps. Curr. Biol. 22, 104–196. 10.1016/j.cub.2012.02.018 22440804

[B36] Therien A. G. Blostein R. (2000). Mechanisms of sodium pump regulation. Am. J. Physiol. Cell Physiol. 279, 541–566. 10942705 10.1152/ajpcell.2000.279.3.C541

[B37] Thompson S. M. Prince D. A. (1986). Activation of electrogenic sodium pump in hippocampal CA1 neurons following glutamate-induced depolarization. J. Neurophysiol. 56, 507–522. 2428952 10.1152/jn.1986.56.2.507

[B38] Van Essen D. C. (1973). The contribution of membrane hyperpolarization to adaptation and conduction block in sensory neurones of the leech. J. Physiol. (Lond.) 230, 509–534. 4717151 10.1113/jphysiol.1973.sp010201 PMC1350612

[B39] Wit A. L. Cranefield P. F. Gadsby D. C. (1981). Electrogenic sodium extrusion can stop triggered activity in the canine coronary sinus. Circ. Res. 49, 1029–1042. 10.1161/01.RES.49.4.1029 7273354

[B40] Xie Z. Cai T. (2003). Na+/K+-ATPase-mediated signal transduction: from protein interaction to cellular function. Mol. Interv. 3, 157–168. 10.1124/mi.3.3.157 14993422

[B41] Zhang H.-Y. Sillar K. T. (2012). Short-term memory of motor network performance via activity-dependent potentiation of Na^+^/K^+^ pump function. Curr. Biol. 22, 526–531. 10.1016/j.cub.2012.01.058 22405867

